# Advancing the STMS genomic resources for defining new locations on the intraspecific genetic linkage map of chickpea (*Cicer arietinum *L.)

**DOI:** 10.1186/1471-2164-12-117

**Published:** 2011-02-17

**Authors:** Rashmi Gaur, Niroj K Sethy, Shalu Choudhary, Bhumika Shokeen, Varsha Gupta, Sabhyata Bhatia

**Affiliations:** 1 National Institute of Plant Genome Research, Aruna Asaf Ali Marg, Post Box No. 10531, New Delhi 110067, India; 2 Defence Institute of Physiology and Allied Sciences (DIPAS), Defence Research and Development Organization (DRDO), Timarpur, Delhi 110054, India; 3 Department of Biotechnology, Chhatrapati Shahu Ji Maharaj University, Kanpur, Uttar Pradesh, India

## Abstract

**Background:**

Chickpea (*Cicer arietinum *L.) is an economically important cool season grain legume crop that is valued for its nutritive seeds having high protein content. However, several biotic and abiotic stresses and the low genetic variability in the chickpea genome have continuously hindered the chickpea molecular breeding programs. STMS (Sequence Tagged Microsatellite Sites) markers which are preferred for the construction of saturated linkage maps in several crop species, have also emerged as the most efficient and reliable source for detecting allelic diversity in chickpea. However, the number of STMS markers reported in chickpea is still limited and moreover exhibit low rates of both inter and intraspecific polymorphism, thereby limiting the positions of the SSR markers especially on the intraspecific linkage maps of chickpea. Hence, this study was undertaken with the aim of developing additional STMS markers and utilizing them for advancing the genetic linkage map of chickpea which would have applications in QTL identification, MAS and for *de novo *assembly of high throughput whole genome sequence data.

**Results:**

A microsatellite enriched library of chickpea (enriched for **(**GT/CA)_n _and (GA/CT)_n _repeats) was constructed from which 387 putative microsatellite containing clones were identified. From these, 254 STMS primers were designed of which 181 were developed as functional markers. An intraspecific mapping population of chickpea, [ICCV-2 (single podded) × JG-62 (double podded)] and comprising of 126 RILs, was genotyped for mapping. Of the 522 chickpea STMS markers (including the double-podding trait, screened for parental polymorphism, 226 (43.3%) were polymorphic in the parents and were used to genotype the RILs. At a LOD score of 3.5, eight linkage groups defining the position of 138 markers were obtained that spanned 630.9 cM with an average marker density of 4.57 cM. Further, based on the common loci present between the current map and the previously published chickpea intraspecific map, integration of maps was performed which revealed improvement of marker density and saturation of the region in the vicinity of *sfl *(double-podding) gene thereby bringing about an advancement of the current map.

**Conclusion:**

An arsenal of 181 new chickpea STMS markers was reported. The developed intraspecific linkage map defined map positions of 138 markers which included 101 new locations.Map integration with a previously published map was carried out which revealed an advanced map with improved density. This study is a major contribution towards providing advanced genomic resources which will facilitate chickpea geneticists and molecular breeders in developing superior genotypes with improved traits.

## Background

Molecular genetic maps covering extensive parts of the genome are essential tools for genomics research, throwing light on genome organization, facilitating marker-assisted breeding of agriculturally important quantitative and qualitative traits and map-based cloning of important genes. Currently the co-dominant microsatellite based STMS markers remain a standard for the construction of highly saturated linkage maps in several economically important crop plants such as wheat [1], barley [2], maize [3], tobacco [4], sunflower [5], rose [6], apple [7], tomato [8] and legumes like soybean [9,10] and peanut [11].

Even though considerable progress has been achieved in many crops for studying the genetics of quantitative traits, in the 2^nd ^(after bean, based on harvested area) most important grain legume crop i.e. chickpea (*Cicer arietinum *L.; 2n = 2x = 16) (FAOSTAT 2009; http://faostat.fao.org/site/567/default.aspx), genomics-assisted programs have moved at a slow pace. The crop has a genome size of 740 Mb and is primarily cultivated in arid and semi-arid areas of the world. Despite it being a protein-rich food, the current average yield of chickpea is only 798 Kg/ha which is far below the potential yield of 6.0 t/ha and is relatively low as compared to pea (1,468.7 Kg/ha) (FAOSTAT 2009). Susceptibility of the chickpea crop to various biotic and abiotic stresses and the low levels of genetic variability are the major constraints to its improvement [12,13]. Moreover, owing to the extremely low levels of genetic polymorphism [14,15], progress towards the development of a sufficient number of polymorphic markers has been limited. Therefore in order to reap the benefits of enabling biotechnologies for crop improvement, there is a pressing need to increase the availability of genomic resources which serve as tools to assist in plant breeding programs. Hence, the central goal of current chickpea researchers is to enrich genomic resources such as molecular markers, especially SSRs, and genetic linkage maps, comprising loci of both economic and scientific importance [13].

Among the vast repertoire of molecular markers currently available, STMS markers have emerged as the best tool to address the allelic diversity in chickpea [16-19]. Further, owing to their ability of interspecific transferability, STMS markers have been reported to be the most elite anchor markers for merging different genetic maps and for setting up a high genome coverage consensus map in chickpea [13,20]. Unfortunately, unlike other legumes like *Medicago *and soybean, till date in chickpea only about 800 STMS markers have been reported [16,18,21-26], and of these only 30-40% are expected to be polymorphic. Nevertheless, microsatellites which are known to be abundant and uniformly distributed in the chickpea genome have been used to develop a genotyping kit for chickpea [19], analyze genetic relationships among *Cicer *species [23,27] and assess levels of cross-transferability [28,29]. Further, these markers have been applied for the construction of intraspecific [30-36] and interspecific [21,26,37-39] genetic linkage maps and for mapping genes of agronomic importance such as disease resistance [37,39,40] and yield related traits [30,41,42], thereby demonstrating that SSRs are ideal tools for broad applications in basic and applied plant biology [43,44]. However, all these studies have repeatedly used only the limited set of available STMS markers and not more than 120 STMS markers have been mapped on the intraspecific linkage maps currently available [30,34,35]. Hence these maps have been of limited use as genomic regions harboring genes of important traits are not yet sufficiently saturated to apply MAS in plant breeding programs. Therefore, the immediate need to map new genomic locations and merge different genetic maps to saturate the intraspecific maps for uniform genome coverage was clearly evident.

Hence the present study was undertaken with the objective of developing a large number of STMS markers which could be utilized by the chickpea community for various applications in chickpea genomics. Next, these markers along with the other published STMS markers were used to advance the intraspecific genetic linkage map of chickpea by defining many new genomic locations. Finally, data of already published loci was integrated with our map to further saturate genomic regions.

## Results

### Characterization of microsatellites and development of STMS markers

Four thousand recombinant clones from the (GT/CA) and (GA/CT) microsatellite enriched library were screened which resulted in the identification of 387 clones that were sequenced. Assembly yielded a set of 22 contig and 314 singleton DNA sequences which summarized a total of 336 unique chickpea sequences. SSR mining revealed that 37 of these either contained an SSR sequence of <5 repeats or did not contain any microsatellites. Moreover, primers could not be designed against 45 of the sequences due to insufficient length of SSR-flanking sequences. Ultimately, 254 (75.5%) primer pairs were designed that flanked the microsatellite motifs. All these primer pairs were validated by PCR using genomic DNA from a set of four *C. arietinum *accessions. Of these, 48 (18.8%) primer pairs produced no PCR products under a number of annealing/elongation temperature combinations, 25 (9.8%) amplified anomalous fragments and 181 yielded fragments of expected sizes. The sequences of these 181 functionally validated primers and the respective microsatellite motifs are listed in Table 1.

**Table 1:** List of 181 novel chickpea STMS markers developed in this study; the locus name, type of repeat motif, primer sequences, annealing temperature (T_m_), expected product size (bp), number of amplified alleles (N_a_), and GenBank accession numbers are mentioned.

**S. No**.	Locus	Repeat motif	Primer sequence (5'→3')	**T**_**m **_**(**^**0**^**C)**	Size (bp)	**N**_**a**_	**GenBank Acc. No**.
1	NCPGR101	(CT)_18_	TCTGCTCTTTGTGCAGAAGAAT/GAAATAATGCGTTCACTGTTG	59.3	291	1	EU877268
2	NCPGR102	(CA)_12_N_19_(CA)_13_	GCGTGGACTAACATCCAATA/TAAAAACATTGGTGGCAACT	55.4	240	1	EU877269
3	NCPGR103	(CT)_2_tc(CT)_21_	ACAACCATATACTTTTGGCG/TTAGATGAAAAACGGGAGAA	55.0	213	1	EU877270
4	NCPGR104	(GA)_21_	GCTAAAGGTAGATATGGGCA/GTGGACTACTCGGAATTCAT	54.3	221	1	EU877271
5	NCPGR105	(CT)_16_at(CT)_7_at(CT)_3_at(CT)_3_at (CT)_3_at(CT)_3_at(CT)_18_	TTTTTGTTAAGCCATCAAAGT/TTTCCCTTTTAGAATGATGC	54.5	261	1	EU877272
6	NCPGR106	(GA)_39_	ATTTGCCTTACATGGTGATT/ATTTGCTTTTCCTTTTCAGA	54.5	229	1	EU877273
7	NCPGR107	(CT)_22_	AAACTCAATATTGCCCTTCA/CCATAACTGGATTGAGCTTT	54.0	244	1	EU877274
8	NCPGR108	(CT)_20_(GT)_16_	AGTTCAAGCCTCATTGATGT/TGAAGAAGAATGGAGAAGGA	54.5	278	1	EU877275
9	NCPGR109	(CT)_12_cccc(CT)_10_	TAGCTCAAAGAGATAACCCG/AAAACAAATCACCTACCCCT	55.1	285	1	EU877276
10	NCPGR110	(AT)_6_(GT)_4_gc(GT)_32_at(GT)_5 _ct(GT)_10_	CAAGGTCAATTCGTAGAAGG/GAACGAGAGTTGGTATTGTTG	55.2	217	2	EU877277
11	NCPGR111	(CT)_22_	AATAACTCCATTTGGCTTGA/GCGGTAATTACACAATACAGG	54.5	247	1	EU877278
12	NCPGR112	(CA)_9_cg(CA)cg(CA)cg (CA)_12_	TTTTATTTCTCACCCACCAG/TGAGTTGCAACGAGAGTAGA	54.5	290	3	EU877279
13	NCPGR113	(CT)_5_ca(CT)_17_(CA)_7_ct(CA)_5_	ATTCTCTCTCTCTCTCTCTCTCGTG/CGGTAACATTCTCAACGGATA	58.0	299	1	EU877280
14	NCPGR114	(GA)_3_gg(GA)_19_	TAAGAGGGGACTTCACATTG/GCGTGGACTAACTACACCAG	55.0	279	1	EU877281
15	NCPGR115	(CT)_18_	TGGAGCCCAATTGATAGCTT/TGGACTACTCGCATTGTTGC	60.2	213	1	EU877282

16	NCPGR116	(GA)_21_	ATTTCCTTTCTTTACGGGAC/AGCGGATAACAATTTCACAC	55.4	295	1	EU877283
17	NCPGR117	(CT)_23_	GAACTTCTTCAATCTCACGG/CTAGCACGATGAAAGGATTC	54.5	199	1	EU877284
18	NCPGR118	(GT)_12_(GA)_18_	GAGTCGATTTCGTGTTGATT/ACGTGAAATTCCACCACTAC	55.5	224	1	EU877285
19	NCPGR119	(CT)_8_N_10_(CT)_19_	GTGGCTGCCTTTTCTTTCAA/TCAAAATACACCGGGGCTAA	60.1	234	1	EU877286
20	NCPGR120	(GT)_20_	GCCCAGTTTTTGGTATTTAG/TATGTTCTTTCTCACCCACC	54.7	300	4	EU877287
21	NCPGR121	(GT)_4_N_8_(GA)_15_	TGATTGTGGGGAACAGAAAT/TGTTGTTTGAAGTTCCGACTG	58.9	215	1	EU877288
22	NCPGR122	(GA)_15_g(GA)_2_(GA)_8_aa(GA)_5_	TGTTCTTTGGCTTGATTTCT/TTGTGAGGATAAGAACGACC	55.0	289	2	EU877289
23	NCPGR123	(CT)_25_	CTCTGCAGACTGAGGGTAAG/TCTGGAGGAGAAGAGACAAA	55.0	273	1	EU877290
24	NCPGR124	(CT)_20_	TTTGTAACTGATGAGTCCGC/ACTACAAGTTTGGACGAAGG	54.3	140	1	EU877291
25	NCPGR125	(CT)_25_	CGGTTTTGTGTATGGTGAGT/GCATACCATTGTCAACCATT	55.5	169	2	EU877292
26	NCPGR126	(CT)_10_N_21_(CT)_12_t(CT)_3_	AGAAGTGGGGACAAACCTTG/TGTGCATACCATGATTCTTCTG	59.1	324	1	EU877293
27	NCPGR127	(GA)_18_	CATAATGCAAGGGCAATTAG/CTCTTATCTTCATGTTGCCG	55.5	279	1	EU877294
28	NCPGR128	(CA)_9_cg(CA)_2_(CGCA)_4 _(CA)_2_N_42_(CG)_4_(CA)_9_	GCAATGAGCAACTTTTCCTT/ATTGGTGTAACTTTTCCGCT	56.2	290	3	EU877295
29	NCPGR129	(GT)_21_	ACGAAGAATTTAATACCGGA/GAGATTTGAGTTTGACGGTT	54.5	293	2	EU877296
30	NCPGR130	(CT)_24_tt(CT)_2_	GATACTGGTGGAAAAATGGA/CAAGCTCTTTCAGAATTTGC	55.5	245	1	EU877297
31	NCPGR131	(GA)_18_ta(GA)_3_aa(GA)_3_	CTATGCGAGGATTTCTCATC/ATACTCGGCAGACATCTGTT	54.3	290	1	EU877298
32	NCPGR132	(GT)_13_(GA)_25_	GAAGATCTCCGACGATGATA/CGGGGACTAACAAGTGTATG	55.5	242	1	EU877299
33	NCPGR133	(CT)_19_	TGAGTGAAAGGTGGAAAAGA/AAGTTCACCTACCAATGCAA	55.5	265	2	EU877300
34	NCPGR134	(GT)_14_(GA)_22_	CATCCTATGAGAGTTGTCCTCTT/TGTCTTTTTCACACTCTCTCTCTCTC	57.6	250	1	EU877301

35	NCPGR135	(CA)_4_cg(CA)_5_(CG)_2_(CA)_5 _(TA)_5_	GAGGAAACATTTCCGATTTC/TATGCTAATTGAATAGCGGC	55.5	234	1	EU877302
36	NCPGR136	(GT)_7_gc(GT)ac (GT)gc(GT)gg(GT)_10_	GGACTGAGTGAGTTCGTCTT/GTATCCTCGGTTTCCCTATC	54.0	132	2	EU877303
37	NCPGR137	(GT)_6_ct(GT)_3_ct(GT)_3_gg(GT)_5_	GTGATGCGACCATGTGAAAA/CGTGGACTAACACATGAGGA	58.0	287	1	EU877304
38	NCPGR138	(CT)_2_cc(CT)_24_ccc(CT)_4_	ATTCCAAATTGCTGTTGTTG/TGTGGATTTTAGTTGCAATG	54.5	213	1	EU877305
39	NCPGR139	(GA)_40_	TGGGTCTTATTGGGTTTGAT/CATGCATTTAGGATGAACCA	56.5	245	1	EU877306
40	NCPGR140	(GT)_14_gc(GT)gc(GT)gc (GT)_10_	ATTGGTTTGAGAAGTGATGG/TTTTATTTCTCACCCACCAG	55.0	264	2	EU877307
41	NCPGR141	(GA)_8_aa(GA)_13_aa(GA)_9_	ACTCAAAAGACAGCAAAGCA/AGCTTAGAGCACTCACATGC	55.5	211	1	EU877308
42	NCPGR142	(CT)_24_	TAACTCCATTTGGCTTGAGA/TAACCTTATATGGTAGGCGG	54.5	263	1	EU877309
43	NCPGR143	(GT)_14_(GA)_22_	TACTTCCCATCCCTCAGTAA/GAGTGAAAAGTTGAAAACGTG	54.5	220	1	EU877310
44	NCPGR144	(GT)_5_g(GT)_5_(GA)_7_	TCTGAACAAGGTTTTCCTCA/TTCATTTGTCCATCAACCTC	55.5	252	1	EU877311
45	NCPGR145	(CT)_5_(CACT)_2_(CT)_10_ca(CT)_4_N_6_(CT)_4_gtca(CT)_11_	CCATATGAAGATATTGTGGCA/ATCATGGCAAGAGGTAGGTC	56.3	316	1	EU877312
46	NCPGR146	(CT)_18_(CA)_12_	AACGTGAAATTCCACCACTA/GAGTCGATTTCGTGTTGATT	55.4	225	1	EU877313
47	NCPGR147	(CT)_24_(CA)_15_	TGTATGAAAACACTTTGACTCATT/CGATGATATTCTCAGCGAAC	55.5	219	1	EU877314
48	NCPGR148	(GA)_12_N_5_(GA)_9_	ACACAAGCCTATGCAATGA/GCTTGAGTTTATGCTTCTGG	55.9	285	1	EU877315
49	NCPGR149	(GA)_27_	TTAAAAATTCAGGGGGCTCA/AACTCACTACCCCTAGTAGCAAA	60.0	202	1	EU877316
50	NCPGR150	(AT)_5_(GT)_16_	GGACCCGACAACACTACTAA/GGGTTAAAGATGTGCCATAG	54.5	287	1	EU877317
51	NCPGR151	(CA)_14_(TA)_9_	AACTCTGTAATTTGCGACCT/GGAAATAACTTGTTGTTGGG	54.5	284	3	EU877318
52	NCPGR152	(GA)_16_	AAGCAGCCTTCTCTCCATCA/CGCGTGGACTAACTCTTGTTT	60.4	221	1	EU877319
53	NCPGR153	(CT)_16_	TGCCTCAAACTCCTACTCAT/AGTGGAGCTAGGGAAATACC	55.6	281	1	EU877320

54	NCPGR154	(CT)_13_N_12_(CT)_4_N_6_(CT)_7_N_8_(CT)_9_	CGCAACTTCAACGTCTCATT/GTGCAAAAGCAAAACTAGGG	58.9	271	1	EU877321
55	NCPGR155	(GA)_18_	GGGAAAAATAATGAGGAGGA/TGGCTCACAATTTTCTCTCT	55.0	281	1	EU877322
56	NCPGR156	(CA)_12_(TA)_5_	CGATTATGTGTCATCCCTTT/ATTTCAACGTCTCAACCATC	55.5	261	1	EU877323
57	NCPGR157	(CA)_16_(TA)_3_	TCCGTAACAGTGATGAACAA/TGGGATTACACTGGATAAGG	55.1	203	1	EU877324
58	NCPGR158	(CT)_3_tc(CT)_14_N_3_(CT)_3_t(CT)_8_	TAAAGCTGGAAACTCGAAAG/TAACCTTCCAATACCGAAGA	55.6	179	1	EU877325
59	NCPGR159	(GT)_9_(GC)_4_(GT)_2_gggc(GT)_3_(GC)_2_N_36 _(GCGT)_4 _(GT)_9_	TGTAACTTTTCCGCTGCTTGT/GGCAATGAGCAACTTTTCCT	59.3	285	1	EU877326
60	NCPGR160	(GT)_12_(GA)_11_	GTGGAGCCAAAAATCGACAT/CGGGCACGAAATATCTGAAG	59.9	241	1	EU877327
61	NCPGR161	(CT)_17_	ACCATCGCAATGCTTTGTTT/CCCTTTTACACAAGGCCAGTAA	60.5	238	1	EU877328
62	NCPGR162	(CT)_17_	GCGTGGACTATTCCTTCAGA/TAGTCGAGGAGTCAATCCGTA	57.8	139	1	EU877329
63	NCPGR163	(GA)_47_	CAAAACTCGCTCGAAACACA/TCCAAACTTTCTCTCTCTCTCTCTC	60.0	164	1	EU877330
64	NCPGR164	(CT)_6_ca(CT)_14_	CCATAACCATAACCCTTTCA/TCTTCTCCTAAGTTGATGGG	54.0	211	1	EU877331
65	NCPGR165	(GA)_15_	TCAGAAGAAAACGAAAGAGC/CAGCAACCTTAATTGGACAC	55.5	233	1	EU877332
66	NCPGR166	(CT)_7_(CA)_11_	TGGATTGTGGTATCCAAAAGG/CAGCATCATCAAAGGTGCAT	59.6	197	1	EU877333
67	NCPGR167	(AT)_5_(GT)_13_	AGATGCAGCGTTTTCCAGAG/CCTTCTTTTTCCTTCCCTTCC	59.7	247	1	EU877334
68	NCPGR168	(GA)_31_	TCCAATACCGAAGAGGCTCA/CGCGTGGACTAACGATTAACA	60.4	243	1	EU877335
69	NCPGR169	(CT)_5_(CACT)_2_(CT)_10_ca(CT)_4_N_6_(CT)_4_gtca(CT)_11_	CCTCCTTCTTGCTTACAAAG/CATGACAATAATGGTGAACG	54.6	256	2	EU877336
70	NCPGR170	(CT)_18_(CA_)12_	ACGTGAAATTCCACCACTAC/GAGTCGATTTCGTGTTGATT	55.9	224	1	EU877337
71	NCPGR171	(GA)_30_	AAAGACAGCAAAGCAAAGAG/AAAACACCATAAATTCCACG	55.0	205	1	EU877338
72	NCPGR172	(AC)_14_	TTGGTTGGGATTGTTACTTT/TCGCATTCCTAGACAATACA	54.0	300	1	EU877339

73	NCPGR173	(AT)_4_(GT)_12_	AATCTTTGGGGATAAAGGAG/ATGTGACCAAAGTAAGGGTG	54.5	266	1	EU877340
74	NCPGR174	(CA)_11_(TA)_4_	TGAGGGGTTGAGTGAATATC/GTTGGAAATAGTGTCACCGT	54.5	170	1	EU877341
75	NCPGR175	(CA)_19_taca(TA)_8_	AAAACGGGGTTTTACAGAAG/CGATAAAATCACAACCGAGA	56.0	232	1	EU877342
76	NCPGR176	(AT)_6_(GT)_16_	TTGAAAGGTGATGTGGAAAC/GGCAGTAAGGAGAAGAAGGA	56.3	234	1	EU877343
77	NCPGR177	(GA)_19_	GGGGAAAAATAATGAGGAGG/GGCACCCAATTTTCTCTTAC	56.1	253	1	EU877344
78	NCPGR178	(CA)_6_aa(CA)_5_	CCCTTAGATTAGTTGAAACCTG/ACTAACTCCGATGCATTCC	54.5	181	1	EU877345
79	NCPGR179	(CT)_17_	TACCACAAAGCTCTGCCTCCAT/GGAAAAGTGGAGTGGACAACA	62.0	335	1	EU877346
80	NCPGR180	(CA)_4_a(CA)_10_(TA)_4_	TCCGTAACAGTGATGAACAA/TGGGATTACACTGGATAAGG	55.0	283	1	EU877347
81	NCPGR181	(TA)_5_(TG)_6_cg(TG)_6_	GAAATGATGGAAGGTGATGT/AGGTTGGAGGAAGAAGAAAG	54.5	264	2	EU877348
82	NCPGR182	(CA)_12_(TA)_2_	CCCAAAGAAGACAAAACAAC/TCATTTAAGGCAGGTCAGTC	54.5	190	1	EU877349
83	NCPGR183	(GA)_12_ggata(GA)_9_	AAAACATTGGTGGCAACTCC/AGAGTCACACACACACACACACA	60.5	236	1	EU877350
84	NCPGR184	(AT)_6_(GT)_16_	TCACTGTGAAAATAGGAAATTTTA/CAGTGATGAAGCTGTTGTTG	55.5	252	1	EU877351
85	NCPGR185	(CT)_17_cg(CT)_3_	TCATGCATTTAGGATGAACCA/CGAACCCTAATTCTCCGTCA	59.4	242	1	EU877352
86	NCPGR186	(CA)_14_(TA)_5_	GTGCATCCATGGTAAAGATT/AACCAGAGTGTAGCCGAATA	55.0	228	2	EU877353
87	NCPGR187	(CT)_9_atc(CT)_13_	CCTTCACTGTCGGTTATGAT/TAACACAAGCCTATGCAATG	54.5	152	1	EU877354
88	NCPGR188	(TA)_2_tg(TA)_3_(TG)_12_	GTTAATTGAGTTGCGACGAG/TCTGTTTCCTTCCTTTTTCC	56.0	181	1	EU877355
89	NCPGR189	(CT)_9_;(CT)_5 _(CACT)_2_(CT)_10 _ca(CT)_4_N_6 _(CT)_4 _gtca (CT)_11_	TGGCACAATGTATGTATTGAA/ATGGCAAGAGGTAGGTCATA	54.5	297	1	EU877356
90	NCPGR190	(AT)_7_(GT)_13_	CCTTAGTGTATAAACCCGAAAC/GACCTGCTTGAGTTAGACCA	54.5	289	1	EU877357
91	NCPGR191	(TA)_4_(TG)_13_	TGGAATTAGTTGATGTGACAATGAG/ATTTCCCGCGTCTTTGAGAT	60.8	221	1	EU877358

92	NCPGR192	(TA)_3_(TG)_12_tt(TG)_2_	TGGGATTACACTGGATAAGG/TCCGTAACAGTGATGAACAA	55.1	203	1	EU877359
93	NCPGR193	(AT)_9_gtat(GT)_9_	CCGATAAAATCACAACCGAG/AAACGGGGTTTTACAGAAGG	58.3	232	1	EU877360
94	NCPGR194	(TG)_6_g(TG)_5_(AG)_7_	AGCCAAAAATCGACATAGAA/ATTTCATTTGTCCATCAACC	54.5	190	1	EU877361
95	NCPGR195	(CA)_11_ga(CA)_5_ta (CA)_31_cg(CA)_5_(TA)_6_	GGATGAACGAGAGTTGGTAT/CAAGGTCAATTCGTAGAAGG	54.0	221	4	EU877362
96	NCPGR196	(CT)_17_	TTGGGTCATTACCTTCATCT/CTCATCCTTGAGAGAAATCG	54.5	226	1	EU877363
97	NCPGR197	(CT)_17_	AAAGGGATCACAATTCAAAA/TAAAAATCGGGGTGTTACAG	54.5	188	1	EU877364
98	NCPGR198	(GA)_18_	TAGTAGGGGAAATGAAGGTG/GCGTGGACTACTAGCATTAAC	54.0	241	1	EU877365
99	NCPGR199	(GA)_27_	GGACATAGTAATCTCCGCTG/CCAACACCAACACCAACATA	55.5	196	1	EU877366
100	NCPGR200	(GA)_24_	TTCACACAACAACCTTTTCA/GGTGAGTTTCTTTTTCCCTT	55.0	250	1	EU877367
101	NCPGR201	(CT)_13_(CA)_12_	TATGCAAGCAATCCTTTAGC/TCTTTTGGAAACTAAGCCCT	55.5	269	1	EU877368
102	NCPGR202	(CT)_25_	AGGCCTTTTCCTTTTTACCT/GGAAAAATTCCCGATCATAC	56.5	259	1	EU877369
103	NCPGR203	(GA)_31_	GAAGAGTTCTGTTGCGGTAG/ATTGGTAATGGCTCAACATC	55.8	157	1	EU877370
104	NCPGR204	(CT)_7_(CA)_17_	TCTTGCCTTTACGTCGACAA/GAATCGATTAAGAAACGTGTGTG	59.2	181	1	EU877371
105	NCPGR205	(CA)_17_(TA)_5_	AAGCAAAAGGAAGCAAAGAA/AGTGGGTTGAGAAATTACGG	56.5	267	1	EU877372
106	NCPGR206	(GA)_3_ta(GA)_7_aa(GA)_8_	AACAACACTGGGTGAGAGAT/GATCCACATGCTACCATACC	54.3	252	1	EU877373
107	NCPGR207	(CA)_10_(CT)_8_	AGACAGGAGAAATGCTGTGG/GCAATGGATGAATGAAAAGG	57.5	281	1	EU877374
108	NCPGR208	(CT)_24_	AGCAAATATTTTGACCTTACACT/ACAGTTAAAAATTCAGGGGG	54.6	178	1	EU877375
109	NCPGR209	(GT)_3_gg(GT)_5_gg(GT)_2_(GA)_7_	ATTGTTTGTTGGAGTGATGG/CACGGTTTCATTGTCTTGTT	55.5	161	1	EU877376
110	NCPGR210	(GA)_17_	AAGGTAGACGTGTGCGTG/CCTGTTATGGAAGATAGGGC	55.5	224	1	EU877377

111	NCPGR211	(CT)_16_	ATCTTCATGTTGCCGACTCC/GCGTGGACTAACCACAAATTC	60.0	213	1	EU877378
112	NCPGR212	(GA)_7_(GT)_12_	CAGTCACTAAACAAGGACTGC/TCAAATCCCAAAATTGATTC	55.0	190	1	EU877379
113	NCPGR213	(CT)_3_(CA)_12_	TTCATGGATGTAATTCTCCC/CCCCACTATTTTCCACATAA	54.5	220	1	EU877380
114	NCPGR214	(CA)_14_(TA)_5_	ATTTCCCGTGTCTTTGAGAT/GGAATTAGTTGATGTGACAATG	54.5	225	1	EU877381
115	NCPGR215	(CA)_3_N_4_(CA)_5_tt(CA)_4_	GTAGCGTGATGTCCTTTCTC/GGCGACAACAGATACTCTTC	54.5	195	1	EU877382
116	NCPGR216	(CA)_11_tc(TA)_3_	GAGCAAGTGTAAACTAGCAAACT/AGCGGATAACAATTTCACAC	55.4	286	1	EU877383
117	NCPGR217	(TG)_15_	GACTACTTGGAATACGTCGC/CGCGCAGTGATTTAAGCTAT	55.1	171	1	EU877384
118	NCPGR218	(AT)_5_(GT)_11_	TTGCTTCGACACTGTAACAC/GCGTGGACTAACTCTTTTCA	54.5	275	1	EU877385
119	NCPGR219	(CA)_13_(TA)_3_	ATGTGACCAAAGTAAGGGTG/ATAAGTGTAGGGTGTCTCAA	54.5	237	1	EU877386
120	NCPGR220	(GT)_13_(GA)_4_	ACTTCTCTACTCAGCCCCTT/GCCCCTATCTTTCAGACTTT	54.5	255	1	EU877387
121	NCPGR221	(CA)_3_cga(CA)cg(CA)_7_(TA)_4_	CATATGCATCATCTCAACCA/TGTCCTTCGTCTTGTTCTTC	55.0	260	1	EU877388
122	NCPGR222	(CT)_22_	TGGTCTTGATTCTTGTCTGG/GAGCAACAAAGCCACAAATA	56.6	165	1	EU877389
123	NCPGR223	(CA)_16_(TA)_6_	TGGGTTTCTTTTCTTGAAGC/AGTGGGTTGAGAAATTACGG	56.5	267	1	EU877390
124	NCPGR224	(AT)_6_(GT)_14_	TGGAATTAGTTGATGTGACAA/ATTTCCCGTGTCTTTGAGAT	54.7	225	1	EU877391
125	NCPGR225	(CA)_3_a(CA)_12_(TA)_3_	TCCGTAACAGTGATGAACAA/TGGGATTACACTGGATAAGG	55.2	203	1	EU877392
126	NCPGR226	(CT)_17_	GACTGCATGTTTTCTTCTCG/ACCACTTCAAAGCCTATTCA	55.3	205	1	EU877393
127	NCPGR227	(CA)_5_N_10_(CA)_24_(TA)_4_	CATTTACCCTCACTTCCGTCA/TGGTTCAGACATCACACCAAA	59.9	207	1	EU877394
128	NCPGR228	(CT)_8_N_10_(CT)_17_	CAACGGTTAAGAATGTGCAA/GCGTGGACTACTCATGTGTCT	57.0	236	3	EU877395
129	NCPGR229	(GA)_3_ta(GA)_15_	CAAATTTTGCGCTGTTGTAG/ACACCTCATCTCCCTTTGAA	57.9	158	1	EU877396

130	NCPGR230	(GA)_26_	CCTCGATTTAAGAGGAACTCA/TGTGTGAAAACACTTTGACTGA	56.7	242	1	EU877397
131	NCPGR231	(GA)_42_	AACCTCCGTCCACACATTTC/GGTCGAAGCCATTGTTTTGT	59.4	226	1	EU877398
132	NCPGR232	(GA)_34_	GGACCGAATGTCCATAAATC/TCTTTTAGGACCCAATGGAG	56.5	265	1	EU877399
133	NCPGR233	(CA)_17_(TA)_5_	GTTTTTGCGAGGCAGTAAGG/TGAAAGGTGATGTGGAAACG	59.5	243	1	EU877400
134	NCPGR234	(GA)_26_	TTAAAAATTCAGGGGGCTCA/CCCCTAGTAGCAAATATTTTGACC	59.5	188	1	EU877401
135	NCPGR235	(CA)_40_	GACTAACCGCGATCAACACA/TGGTTTGAGAGGTGATGTGG	59.7	182	1	EU877402
136	NCPGR236	(GT)_12_(GA)_25_	CAACGGTAACATTCTCAACG/TTTTCTTTTGATGTGTTCTTGG	56.5	200	2	EU877403
137	NCPGR237	(GA)_2_ta(GA)_24_	ATTGCTCAGCTTTTGGAGGA/CGGGCTGGGAATTAAATAGA	59.9	314	1	EU877404
138	NCPGR238	(GA)_3_a(GA)_18_	GTCCGTGACATTGACACTTT/CATAGTTGGATTGCCTCTCA	56.5	273	2	EU877405
139	NCPGR239	(CA)_4_N_12_(CA)_5_cc(CA)_8_ga(CA)_5_	TGATGAAGGTTGTAAACATGG/GGTGGTTTATGCCACAATAA	56.5	137	1	EU877406
140	NCPGR240	(GA)_17_	AAGGGGTGAGTTTTTGAGTT/CCCCTTAATTTCTTTCTCCA	55.0	238	1	EU877407
141	NCPGR241	(TA)_5_(TG)_15_	GCGTTTTCCAGAGAAATTCA/GGGAGGAAACATTTTCGTTT	58.7	250	1	EU877408
142	NCPGR242	(CT)_11_(CA)_12_	TCGTCATATCCACCCGATAA/TGGATAATGGTGCGAAAGAA	58.5	145	1	EU877409
143	NCPGR243	(CA)_13_	TGCTTGGGCGAGAGTAGTTA/GCGGCGTTTAGTTTCTTCAA	58.7	206	1	EU877410
144	NCPGR244	(CT)_2_c(CA)_11_	TGGACTACTGAATCACTCCCTCT/TGCTAAGTTGTCTGGGTGGA	59.2	200	1	EU877411
145	NCPGR245	(CA)_13_	GTTTGACTAAATATGGGGCA/AAGGATGAGTCATGGAAAAA	54.5	148	1	EU877412
146	NCPGR246	(CA)_13_	GTGGACTAACCCACATAGGA/ACCATTACCAGAAACCATGA	54.5	154	1	EU877413
147	NCPGR247	(GT)_12_	CAATGATTGGTTCTCTCCTC/GGTTTGACTAAAATATGGCG	54.5	105	1	EU877414
148	NCPGR248	(GT)_12_	GGCATTGTATGGAAGGAGGA/CGCGTGGACTACCATATCATT	59.8	230	1	EU877415

149	NCPGR249	(CA)_5_a(CG)_3_(CA)_10_	CTCTTCGATTCGGATAGGTT/TGTTTTCAGCTAAATTTCACG	55.5	231	1	EU877416
150	NCPGR250	(CA)_10_	CGCGTGGACTAACTTCTGTA/TGGCCTAACAGCTTTCCATT	57.9	243	1	EU877417
151	NCPGR251	(CA)_13_	AATGGGTTAATTTGACTTGC/TTAATGGCCACCATAATCTT	54.0	282	1	EU877418
152	NCPGR252	(CA)_12_	TTGCCCTGAGGAATACATTA/GGTTGTTGAAGGCATAACTG	54.3	187	1	EU877419
153	NCPGR253	(GT)_12_N_21_(GT)_21_	ACATTGGTGGCAACTCCATT/GGCGTGGACTAACATCCAATA	60.0	236	1	EU877420
154	NCPGR254	(AT)_2_(GT)_11_	GCCTTTTTCAATTTCTCTCA/CCCAAAGAAGACAAAACAAC	54.5	298	1	EU877421
155	NCPGR255	(GT)_12_	TCAGTGGTATTGAGACATCG/CCATCTTCAAAAGTGAACCT	54.0	258	2	EU877422
156	NCPGR256	(CA)_12_	AATGGGTTAATTTGACTTGC/TTAATGGCCACCATAATCTT	54.2	280	1	EU877423
157	NCPGR257	(GT)_5_gc(GT)_4_	CCAAAGGTGCGATGAAAATC/GCGTGGACTACTCTTCATGT	58.2	182	1	EU877424
158	NCPGR258	(CT)_7_atca(CT)_4_	TTTTACCAATGACTGGCTGA/TTGTGGTGAAGAATCTGAAGAG	56.5	250	1	EU877425
159	NCPGR259	(GT)_12_	TATAGCCATAAGGGCAACAT/TGTGGTAGAATGGGGAATAG	55.6	185	1	EU877426
160	NCPGR260	(GT)_12_	CGGCGTTTAGTTTCTTCAAT/ATTAAGTTGGGTAACGCCAG	56.5	247	1	EU877427
161	NCPGR261	(CA)_2_t(GT)_12_	GATTGTGTGGCAAAATCCAT/ACTCTCAGGTTGCTGTTCTGA	58.9	300	1	EU877428
162	NCPGR262	(GT)_13_	GATAAGCGATAACCTTGTGG/CGCGTGGACTAACATATCAT	55.0	185	1	EU877429
163	NCPGR263	(GT)_10_	CAAGGATGAATGTGTGTGTG/CATAGTATCCTCGGTTTCCC	55.5	111	2	EU877430
164	NCPGR264	(GT)_3_gg(GT)_5_gg(GT)_2_	TGGGAATCTTGTTGGTTCTT/TGAAAGGAGATGGAAAAAGC	57.1	221	1	EU877431
165	NCPGR265	(GT)_11_(CT)_2_	GTGTTTGTTGCTCTGTCTGA/CACCCACACACATACACAGT	54.5	195	1	EU877432
166	NCPGR266	(CA)_12_	TGTGAAAACTGATGAGGACA/GTGTGTTGTCGTTTGTCTTG	54.5	195	1	EU877433
167	NCPGR267	(TA)_2_(CA)_13_	ATTAACTGTGCTGGAGGAAA/TATAGCCATAAGGGCAACAT	54.5	279	1	EU877434

168	NCPGR268	(GT)_11_	TCAACTAAGGATTTGCTCG/AGAGCTGAGAGAGTGGACAA	54.5	296	1	EU877435
169	NCPGR269	(GT)_9_	CGTGGAACTATCGAAAGGTGT/ATAAGCCAAGGGAGGACGAA	60.5	221	1	EU877436
170	NCPGR270	(GTATGTAT)_2_(GT)_10_	GTTTGTAAGAACTGAAAAGTTGTGC/CGTGGACTAACCCACATAGGAAT	60.0	236	1	EU877437
171	NCPGR271	(CA)_13_	TGGAATTAGTTGATGTGACAATGA/CGGAGGGTGAGAAGCAGT	59.1	355	1	EU877438
172	NCPGR272	(AT)_4_(GT)_13_	TGGACTAACAGCTTTCCATT/GTCTTCTGTAGATTGAAGTTGTAAA	54.5	233	1	EU877439
173	NCPGR273	(CA)_11_	CCATCTTCAAAAGTGAACCT/TCAGTGGTATTGAGACATCG	54.6	273	1	EU877440
174	NCPGR274	(GT)_12_	GTGTGTTGTCGTTTGTCTTG/TTTTGAAGAGCAATCAATCC	55.9	268	1	EU877441
175	NCPGR275	(CA)_7_(TA)_5_	CGAGGAAGCATTCTGCATT/TCCTGGAGCCTCGATTAAA	58.0	355	1	EU877442
176	NCPGR276	(CA)a(CA)_9_	CTGCAAAATCGAAGGGAGGT/GCATGCGTCTTTCTCTCTTT	56.9	257	1	EU877443
177	NCPGR277	(CT)_17_	CAGCTACTCCATTATTTTGTGTTT/CACATGAAGTCGTCCAACAA	56.5	278	1	EU877444
178	NCPGR278	(GT)_5_g(GT)_3_gc(GT)_2_	TGAGACATCGACTATTGGACA/GACCATCTTCAAAAGTGAACC	56.0	250	1	EU877445
179	NCPGR279	(CT)_17_cctt(CT)_2_	TTTGAGGTCTTACTCTTTACAGC/ATTAAACGTGAGGGAGAAAA	54.5	248	1	EU877446
180	NCPGR280	(GT)_13_	GCAATGATTGGTTCTCTCCTT/TTTGGGTTTTCTAGCTCCTT	56.5	207	1	EU877447
181	NCPGR281	(GT)_9_	GCAATGATTGGTTCTCTCCT/GTGGAATTCTTTAGGGTTTGAC	56.5	114	2	EU877448

As expected, these 181 SSR containing sequences were rich in (GT/CA)_n _and (GA/CT)_n _motifs and based on the structural organization, the repeat motifs were classified as perfect (72, 39.7%), imperfect (26, 14.3%), compound (45, 24.8%) and interrupted (38, 20.9%). However, the predominance of CA repeats was observed (78 clones; 43.0%) compared to CT repeats (68 clones; 37.5%) while CA and CT compound motifs were found in the remaining clones (19.0%). High variability in the numbers of microsatellite motifs were found at these loci with the maximum number of uninterrupted GA and CA units being 47 (NCPGR163) and 40 (NCPGR235) respectively. However, many long repeat motifs were also present like (GA)_40 _at NCPGR139, and (GT)_20 _at NCPGR120. The longest stretch of compound microsatellite motif was found in NCPGR236 with repeat motif (GT)_12_(GA)_25_. But the majority of the repeat motifs comprised of 12-30 repeat units. 160 primer pairs (83.39%) amplified single alleles whereas, 21 primers (11.6%) produced 2-4 alleles (Table 1). Moreover, with 44 out of the 181 primer pairs, intraspecific variability was clearly detectable among four chickpea accessions even by resolution on simple agarose gel (data not shown).

Similarity search using the BLASTN program at NCBI revealed that the chickpea microsatellite containing sequences had homology with a variety of sequences including repetitive DNA, ribosomal DNA as well as coding sequences of genes and unknown proteins from diverse plant genomes. Forty eight of the sequences were found to be similar to the *M. truncatula *BAC clones whereas 5 sequences showed similarity to known proteins or predicted genes of the same plant. Of the 14 sequences found to be similar to the chickpea genome, only two sequences (NCPGR160, NCPGR164) were similar to the chickpea polypyrimidine track-binding protein (ptb) (AJ549383) and beta-galactosidase genes (AJ012687) respectively, while the remaining sequences were similar to retrotransposons and ribosomal DNA.

### Identification of polymorphic markers and genotyping for linkage analysis

In the present study, a total of 522 chickpea STMS markers (Table 2) including 265 NCPGR series markers developed by us, 150 H-series markers developed by Lichtenzveig et al. 2005 [24] and 107 markers developed and mapped by Hüttel et al. 1999; Winter et al. 1999 [16,21] were used to identify polymorphic primers between ICCV-2 and JG-62, the parental lines of the mapping population. Of the 522 STMS primer pairs, only 226 (43.3%) primer pairs (109 (48.2%) NCPGR series, 69 (30.5%) H-series [24] and 48 (21.2%) of Hüttel et al. 1999; Winter et al. 1999 [16,21]) produced clear and consistent polymorphic banding patterns between the parental lines (Table 2). These 226 polymorphic primers were further used to genotype all the 126 individuals of the RIL population. Genotyping data was obtained for all 226 chickpea STMS markers along with 1 morphological marker (double-podding) and used for linkage analysis.

**Table 2:** Summary of the STMS markers used in the present study for the construction of the intraspecific linkage map of chickpea (*Cicer arietinum *ICCV-2 X JG-62)

	Markers analyzed	Markers polymorphic in parents	Markers mapped No. (%)	Markers distorted
**NCPGR**	265	109	66 (60.55%)	38
**Lichtenzveig et al. 2005 **[24]	150	69	35 (50.72%)	23
**Winter et al. 1999 **[21] **Hüttel et al. 1999 **[16]	107	48	36 (75.00%)	9

**Total**	522	226	137 (60.61%)	70

### Development of an intraspecific linkage map

**Figure 1:**
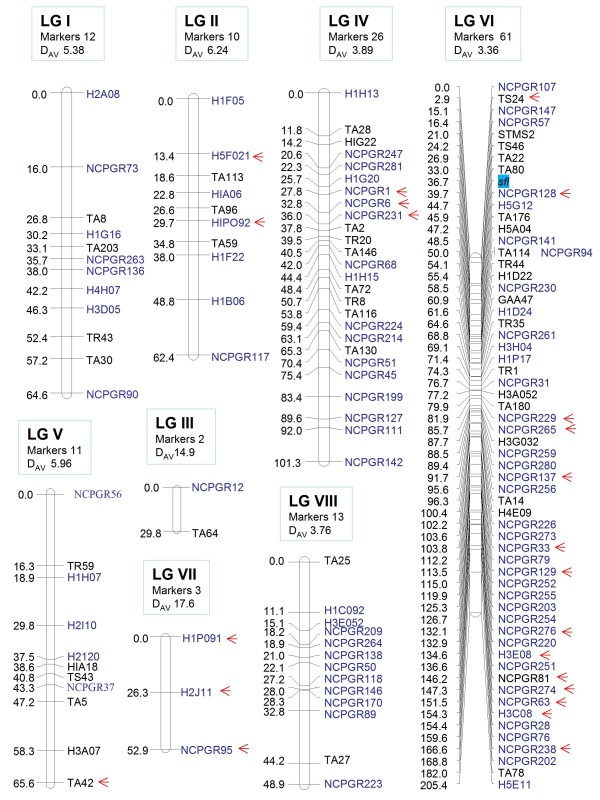
**The intraspecific linkage map of chickpea**. The intraspecific linkage map of chickpea based on RILs of *C. arietinum *(ICCV-2) × *C. arietinum *(JG-62) was generated with STMS markers using JoinMap version 4.0. The name of the linkage groups, the number of mapped markers and the Average Marker Density (D_Av_) is mentioned at the top of each LG. Newly mapped markers (NCPGR-series and H-series) are shown in blue colour and the morphological marker (double-podding, *sfl*) is shown in a shaded box. Arrows represent the markers showing distortion.

JoinMap ver. 4.0 [45] was used to develop the intraspecific genetic linkage map using 227 markers of which 137 STMS and 1 morphological trait (*sfl*) were mapped at a LOD score of 3.5 (Figure 1). The 137 STMS mapped markers included 66 of NCPGR series, 35 of H series [24], and 36 markers of Hüttel et al. 1999 and Winter et al. 1999 [16,21] (Table 2). The current linkage map covered 630.9 cM spanning 8 linkage groups with an average marker density of 4.57 cM (Figure 1). There was a large variation in the lengths of individual linkage groups that varied from a maximum of 205.4 cM to a minimum of 29.8 cM and genome coverage varying from 96.0% (LG6) to 33.0% (LG3). Relative to the estimated physical size of the chickpea genome (750 Mbp) [46], 1 cM distance in the present map approximately equals to 1.18 Mbp.

In order to facilitate comparisons with the previously published studies, the maps of Winter et al. 2000 [37] and Millan et al. 2010 [20] were considered as reference maps and the LGs in our map were named (LGI-VIII) to conform to these maps [20,37] based on the common set of 30 markers present in the LGs (Figure 1). The current map (Figure 1) revealed that the markers were not distributed evenly throughout the genome as some of the linkage groups were densely populated with markers while other LGs were sparsely packed (Figure 1). LGVI was the largest linkage group both in terms of size (205.4 cM) and number of mapped markers (61). It defined new positions of 34 NCPGR series and 12 H-series markers with an average marker density (D_Av_) of 3.36 cM. The double-podding gene (*sfl*) also mapped to this linkage group and was flanked by TA80 and NCPGR128 at 3.7 cM and 3.0 cM respectively. This linkage group shared 8 markers (TA14, TA22, TA176, TA80, TR44, TS24, Tr35 and STMS2) with the corresponding LGVI of the interspecific map [37]. LGV spanned 65.6 cM, harbouring 11 markers and shared 4 common markers (TR59, TS43, TA5, and TA42) with LGV [37]. LGIV was composed of 26 loci containing 14 NCPGR series and 4 H-series markers spanning 101.3 cM with average marker density of 3.89 cM and contained 5 common STMS loci namely TR20, TA2, TA72, TA130 and TA146 with LGIV of Winter et al. 2000 [37]. LGVIII was one of the smallest linkage group, having marker density of 3.76 cM and defined positions of 9 NCPGR series markers. LGI spanned 64.6 cM with 12 markers mapped at an average marker density of 5.38 cM and corresponds to LGI [37] as they shared 3 loci namely TA8, TR43 and TA203. LGII had 10 markers and shared 2 common markers (TA59 and TA96) with LGII [37]. LGVII spanned 52.9 cM and had an average marker density of 17.6 cM, but did not possess any common markers from Winter et al. 2000 [37]. LGIII was the smallest linkage group spanning 29.8 cM that housed only 2 markers, one of which (TA64) was common with LGIII of Winter et al. 2000 [37]. The wide range of marker density (3.36 in LGVI to 17.6 in LGVII) indicated differing degrees of saturation of linkage groups with the new set of markers.

Of the 226 STMS markers analyzed, 70 (31.0%) markers did not segregate according to the expected Mendelian ratio. Out of these 70, the majority of markers (43; 61.4%) showed slight deviation from the ratio while 27 loci (38.5%) exhibited significantly high segregation distortion. Further, analysis revealed that the frequency of distorted female markers appeared to be double (43 markers; 61.4%) as compared to distorted male markers (27 markers; 38.6%). Of 70 loci, 23 (32.8%) markers were mapped and most of them resided on LGVI and LGVII and were indicated by arrows on the linkage groups (Figure 1).

### Map compilation and integration

**Figure 2:**
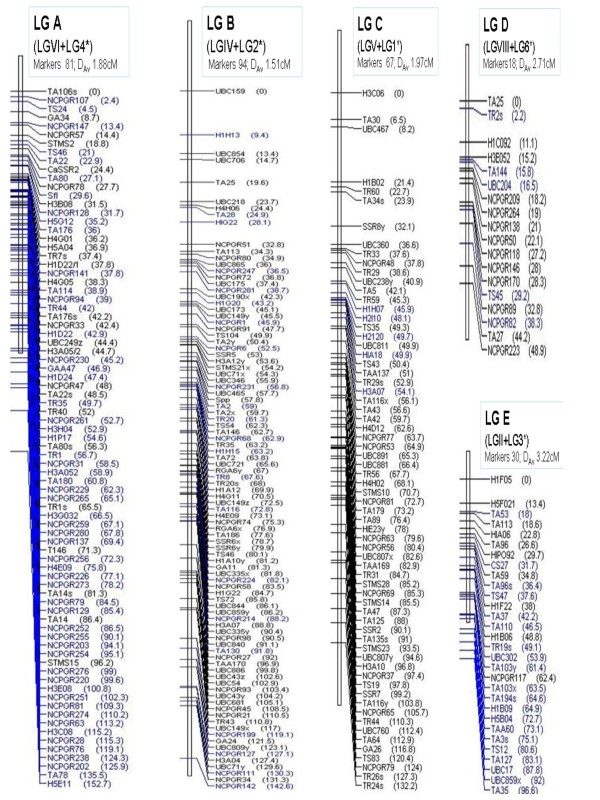
**Map of projected linkage groups**. Markers from the LGs of 2 maps namely ours (from Figure 1) and Radhika et al. 2007 [34] (marked by *) were combined to obtain the 5 projected LGs (designated A-E). The software BioMercator ver. 2.1 [47] was used for the integration of the individual LGs. Markers shown in black colour are from the map of Radhika et al. 2007 [34] whereas markers from our map (Figure 1) are in blue. Total number of markers and the Average Marker Density (D_Av_) is mentioned above each LG.

Comparison of our map with the recently published intraspecific map of chickpea [34] was carried out. Since the LGs in Radhika et al. 2007 [34] were not named according to Winter et al. 2000 [37], hence 47 common markers between our map (Figure 1) and that of Radhika et al. 2007 [34] were identified which were distributed across five LGs. Hence five of our linkage groups namely LGII, LGIV, LGV, LGVI and LGVIII were integrated with the corresponding LG3, LG2, LG1, LG4, and LG6 respectively of Radhika et al. 2007 [34] using the program BioMercator ver. 2.1 [47]. The map of the 5 compiled LGs (designated LGs A-E; Figure 2) illustrated that even though the overall map lengths of the projected LGs remained almost same but the marker density improved dramatically. For example, after combining our LGVI (61 markers) with LG4 [34] (26 markers) the inter-marker distance improved to 1.88 cM from 3.36 cM (LG A; Figure 2). This combined LG A clearly helped in fine mapping of *sfl *region such that flanking markers TA80, NCPGR78, H3B08, and NCPGR 128 which have been shown to be closely associated with the s*fl *gene in the previous maps [34] and in our map (Figure 1), now position more closely at a distance of 2.5 cM, 1.9 cM, 1.9 cM, and 2.1 cM respectively from the *sfl *region. Remarkable improvement was also obtained when our LGIV was combined with LG2 of Radhika et al. 2007 [34] (72 markers) to accommodate 94 positions with marker density of 1.51 (LG B; Figure 2). Similarly, projections of our LGII, LGV and LGVIII on LG3, LG1 and LG6 of Radhika et al. 2007 [34] respectively, substantially improved the marker densities of each of the LGs (LGs C, D, E; Figure 2).

## Discussion

Availability of the chickpea genomic resources is still in its infancy. Most imperative among these are the SSR markers, ESTs and a saturated linkage map. A critical mass of polymorphic SSR markers is still limited in chickpea as only about 800 have been reported till date [16,18,21-26] of which only about 30% are expected to be polymorphic. Hence, keeping in mind the limited number of available SSR markers coupled with the low levels of polymorphism in chickpea, it was necessary to generate several additional SSR markers which could be used to construct high-density genetic linkage maps of chickpea. Although several intraspecific linkage maps are available for chickpea with various mapping populations [20,30,33-36], all these maps have been constructed employing only the STMS markers reported in earlier [16,21] as well as later studies [18,24]. Therefore, as expected, all these maps have exhibited similar genomic locations and similar marker order, and are therefore of limited use. Thus, the primary goal of the present study was to generate new STMS markers and use them to construct an intraspecific genetic linkage map of chickpea to decipher new unmapped regions of the genome. Moreover the integration of this genomic information with a recently available intraspecific map was done to substantially increase the marker density, thereby facilitating the saturation of the linkage map.

The important contribution of the present study was the development of a major genomic resource comprising of 181 genomic STMS markers developed from the microsatellite enriched library of chickpea. Use of this enrichment method [48] significantly increased the efficiency of SSR marker development since about 10% of the recombinants contained SSR motifs in agreement with earlier reports [48,49]. Moreover a very stringent criterion was used to select the SSR motifs against which STMS primers were developed. Most of the SSRs selected belong to the class I type [50] which include SSRs greater than 20 bp in length and are therefore more polymorphic and more useful as genetic markers. This was clearly evident from the data of polymorphism analysis (Table 2) which showed that 41.1% of our markers (NCPGR series) were polymorphic. Hence the developed STMS markers provide a resource which in future may be utilized for the analysis of genetic diversity, map integration and QTL analysis.

Another achievement of this study was the advancement of the linkage map. Not only were the newly developed 181 STMS markers used for map generation, but 341 additional STMS markers, reported earlier but mostly unmapped, were also used (Table 2). Hence, a total of 522 microsatellite markers were used to screen for polymorphism between ICCV-2 and JG-62, the parental lines of the intraspecific RIL mapping population, and this revealed 226 (43.3%) polymorphic markers. This level of polymorphism was fairly high for a crop like chickpea which has a narrow genetic base and was comparable with earlier studies in chickpea which reported 30-40% polymorphism between the parental lines of the various intraspecific mapping populations [31,32,34,35].

The present linkage map defined 138 map positions which were distributed non-randomly and unevenly over 8 linkage groups. The map spanned 630.9 cM which was comparable with the previous map (739.6 cM) [34]. The map length was larger than the other intraspecific maps (426.99 cM) [20], (534.4 cM) [33], (419 cM) [32], (318.2 cM) [31], (419.7 cM) [36] but smaller than the map (1285 cM) reported by Taran et al. 2007 [35]. Several factors, including population size and the nature and number of markers used in the analysis, may contribute to the difference in map coverage on different populations. Moreover, differences in linkage intensities among different crosses might be responsible for differences in the map coverage [51]. A remarkable feature of this map was the 101 new genomic locations that were defined in this study (which included 66 NCPGR series and 35 H-series markers) in the backdrop of the previously mapped STMS markers [37]. These new locations would be beneficial to chickpea breeders to tag important genes and QTLs. Even though the number of linkage groups defined in this study were the same as expected for chickpea haploid number (n = 8) the density of the markers indicated the need to add more markers to the small groups which would then coalesce and be integrated to construct the detailed genetic linkage map.

About 31.0% of markers used for linkage analysis did not follow the expected Mendelian ratios. This could be compared with the studies [34,37] in chickpea and with other plant species such as *Arabidopsis *[52], rice [53] and *Medicago *[54-56]. From the genetic mapping projects, it is clear that variations from expected Mendelian ratios are common within both interspecific and intraspecific crosses [54], however generally higher percentage of allelic distortion was observed in the former case. Hence, the mapping of new STMS markers on the intraspecific genetic linkage map was preferred as it would serve chickpea breeders more accurately than interspecific maps by alleviating problems like marker distortion [30,33]. In tomato, Paran et al. 1995 [57] reported a significant increase in the number of loci that deviated from the expected Mendelian inheritance from F_2 _to F_7_. They accounted this increase to the cumulative effect of selection against the alleles of one of the parents during propagation of the RILs. A similar level of segregation distortion was also reported for mungbean from F_3 _to F_7 _population [58,59]. Interestingly, the distorted markers in the present map were majorly concentrated on linkage groups VI and VII suggesting that some structural reasons might be responsible for this distortion. Moreover, most of the distorted loci (61.4%) were skewed in favour of the maternal alleles i.e. JG-62. This might be due to accumulation of distorted alleles in the population with progressive cycles of selfing undergone in the development of the RILs [33].

In the current map non-random distribution and clustering of markers was observed for most linkage groups leading to large variations in the marker density. This might be attributed to the fact that microsatellite sequences in the chickpea genome may cluster around centromeres [60]. Similar clustering of microsatellites around the centromere has been observed in various plant species like sugarbeet [61], barley [62,63], tomato [64,65] and several other *Triticeae *species [63]. Several factors are responsible for this clustering of genomic SSRs on genetic linkage maps, major being their non-random physical distribution in plant genomes [66,67], reduced recombination in centromeric regions [68,69] and the genomic origin of DNA sequences used for SSR development [70].

Currently, the primary goal in chickpea research programs worldwide is to generate the consensus linkage map and to increase the marker density i.e. to place as many markers as possible into a single map. Comparison of the present intraspecific map of chickpea (Figure 1) with the interspecific map developed by Winter et al. 2000 [37] and the consensus map of Millan et al. 2010 [20] revealed high linkage conservation in at least 6 linkage groups and hence we were able to designate our LGs in accordance with these maps. However, the map distances and marker orders of the common SSR markers differed, possibly due to the intraspecific nature of our mapping population. Nevertheless, by developing separate intraspecific maps for *C. arietinum *and *C. reticulatum *using common STMS markers and comparing the map positions might provide the molecular insight into the chromosomal rearrangement events and evolution of chickpea from its wild progenitor *C. reticulatum*. In this context, it was felt that map comparisons and integration with existing intraspecific maps would be more significant. Therefore an effort has been made in the present study to integrate the available information from the intraspecific maps in order to construct a more dense and saturated linkage map of chickpea. The program BioMercator [47] allows merging different individual genetic maps even without the availability of raw genotyping data. Considering the common loci as bridges between maps, this program provides the possibility of building the compiled map by iterative projection. Since common markers were identified on 5 LGs of our map and the recently reported map [34], it was possible to combine these data using the program BioMercator ver. 2.1 [47] (Figure 2). Five highly resolved LGs (LG A-E; Figure 2) were generated with substantially improved marker densities. Such marker densities are highly desirable as they make application of MAS and map-based cloning possible. Also, highly dense maps are now proving useful for de novo sequence assembly of next generation whole genome sequence data by facilitating the anchoring and orienting of the scaffolds [71].

The double-podding gene (*sfl*) which mapped on LGVI in our present map (Figure 1) was flanked by Ta80 and NCPGR128 at 3.7 cM and 3.0 cM respectively (Figure 1) and is known to have a positive yield stabilizing effect and it is independent of seed size [72]. Map compilation helped in saturating this region (LG A; Figure 2). Ta80 which had been earlier shown to be 4.84 cM from *sfl *[41] and 3.7 cM in our map (LG I, Figure 1), now in the projected LG A (Figure 2) was only 2.5 cM apart. Moreover the marker NCPGR78 was embedded between *sfl *and Ta80. In LGI (Figure 1) *sfl *was flanked by NCPGR128 at 3.0 cM which in LG A (Figure 2) reduced to 2.1 cM and accommodated 1 marker (H3B08) between them. Therefore it was clear that the compiled map would serve as a highly useful resource for future mapping projects.

## Conclusions

In the present study, we enhanced the marker repertoire in chickpea by developing a set of 181 novel STMS markers from a microsatellite-enriched library, thereby providing researchers with advanced genomic resources for genomics-assisted breeding programs. To apply the developed resource in breeding, an advanced intraspecific genetic linkage map of chickpea was constructed. New genomic locations were mapped by utilization of new as well as the previously developed but unmapped STMS markers. Marker density was substantially improved by merging the map data generated in this study with the available intraspecific map. Therefore this study will be directly useful in promoting future mapping projects, for dissection of complex agronomic traits and for anchoring and orienting the scaffolds required for assembly of next generation whole genome sequence data.

## Methods

### Plant material and DNA isolation

The intraspecific mapping population of chickpea was generated at ICRISAT, Patancheru, India by Dr Jagdish Kumar. Briefly, *C. arietinum *cv. ICCV-2 (donor parent, large seeds and single pods) a kabuli variety was crossed with *C. arietinum *cv. JG-62 (recipient parent, small seeds and double podded) a desi chickpea variety. The F_1 _plant was self-pollinated to obtain the F_2 _offspring that were further self-pollinated and advanced by single seed descent for next 10 generations to obtain recombinant inbred lines (RILs). A population of randomly selected 126 individuals was used for linkage analysis and map construction. All the plants were grown at the NIPGR field site. Genomic DNA from fresh leaf tissue of all the 126 RILs of intraspecific population along with the parental lines ICCV-2 and JG-62 was isolated using CTAB method [73]. The quality and quantity of all DNA samples were checked on agarose gels by comparison with known amounts of uncut λ DNA.

### Cloning and characterization of microsatellite rich regions

Nuclear DNA of chickpea cv. Pusa 362 was isolated by using the protocol of Malmberg et al. 1985 [74]. The microsatellite enriched library was constructed [48] for the identification of (GT/CA)_n _and (GA/CT)_n _repeats. Approximately 2.5 ng of microsatellite enriched eluted DNA was cloned into 10 ng of a modified pUC19 vector (pJV1) [48]. After transformation and blue-white selection on IXA (IPTG, X-gal and ampicillin) plates, the white colonies were transferred to Hybond N membrane (Amersham Biosciences, USA) and screened using γ [^32^P]-ATP labelled (CA)_10 _and (CT)_10 _oligonucleotide probes. Plasmid DNA from the recombinant clones producing intense signal after autoradiography were isolated using the alkaline lysis method [75], purified by PEG-precipitation and sequenced on ABI3700 Prism automated sequencer (Applied Biosystems, USA). To reduce the redundancy, DNA sequences were assembled using the CAP3 program (http://pbil.univ-lyon1.fr/cap3.php) [76]. Microsatellite detection was done using the TROLL program [77] where ≥5 dinucleotide and ≥4 trinucleotide motifs were selected. The microsatellite containing sequences were submitted to the GenBank for obtaining the accession numbers (EU877268-EU877448) and also subjected to BLASTN analysis at threshold value of 1E-05 for homology searches.

### STMS marker development and polymorphism analysis

100-150 bp regions flanking the microsatellite motifs were identified for designing STMS primers. Primers were designed using the software Primer 3.0 (http://frodo.wi.mit.edu/primer3/) [78] and the criteria for primer design was as mentioned in Choudhary et al. 2009 [79]. The primer pairs were validated by amplification of the expected sized products from chickpea cv. Pusa362 genomic DNA and designated as NCPGR 101-281 (Table 1). The 181 STMS primers developed in this study (Table 1) along with 84 primers developed earlier in our laboratory (NCPGR 1-100) [18,22], 150 primers of H-series [24] and 107 primers reported in earlier studies in chickpea [16,21] were used for analysis of parental polymorphism (Table 2). All the primers were screened for polymorphism between chickpea accessions ICCV-2 and JG-62, the parental lines of the mapping population. Those that exhibited polymorphism were further used for genetic analysis of all the 126 individual RILs of the mapping population.

### Genotyping, linkage analysis and map construction

Since only microsatellite based markers were used, SSR genotyping was done by PCR amplification of genomic DNA from the 126 RILs and the parents followed by gel electrophoresis. PCR reactions were carried in a 15 μl reaction volume containing 40-50 ng of genomic DNA, Titanium Taq PCR buffer (20 mM KOH, 10.6 mM KCl, 2.3 mM MgCl_2_, 2.5 μg/ml BSA), 0.75 μM of each primer, 0.125 mM of each dNTP, and 0.5 U of Titanium Taq DNA polymerase (Takara, Clontech). The following touchdown amplification profile was used: (i) initial denaturation 94°C 3 min, (ii) 18 cycles of 94°C 50 s, 65°C 50 s [decreasing annealing temperature 0.5°C/cycle], 72°C 50 s, (iii) 20 cycles of 94°C 50 s, 55°C 50 s, 72°C 50 s, and (iv) final extension 72°C 7 min. The amplified products were electrophoresed on 6% polyacrylamide gels or 3% Metaphor agarose gels depending upon the size range, stained with ethidium bromide and analyzed using the gel documentation system. The amplified banding patterns were scored as 'A' for ICCV-2 type banding pattern, 'B' for JG-62 type banding pattern and 'H' for heterozygous loci. Additionally, the RILs were also phenotyped for one morphological trait i.e. double-podding (*sfl*) which is reported to be a monogenic recessive trait [41]. The pod number per peduncle was scored for each of the RILs for three consecutive years (in the chickpea growing season of 2006, 2007 and 2008) at the institute field site.

Each segregating marker was tested for goodness of fit to the expected 1: 1 ratio by *χ*^2 ^test (*p <*0.05). All markers including those with distorted distribution were used for linkage analysis and map calculations performed using JoinMap ver. 4.0 [45]. The markers were classified into linkage groups (LGs) using the minimum LOD threshold of 3.5 and maximum of 5.0 with recombination fraction of 0.4. Kosambi mapping function was used to estimate the map distances [80]. The LGs of the present map were designated with Roman numerals from I to VIII. Genome coverage was calculated according to Chakravarti et al. 1991 [81] i.e. Genome coverage = Map length/{Map length × [No. of loci +1/No. of loci-1]}.

### Map Projection

To build the consensus intraspecific linkage map of chickpea, the program BioMercator ver. 2.1 [47] was used. The program facilitates automatic compilations of several genetic maps by iterative projections of genes, loci and QTLs. Common loci between homologous LGs were compiled to compute specific distance ratios for each interval between two common loci. Using this criteria, LGs of our map were projected on LGs of reported map [34] through this program. Further, to saturate the regions harboring the double-podding (*sfl*) gene, further integration was carried out.

## Authors' contributions

RG, SC, NKS and VG conducted the experimental work. RG, SC and SB compiled and analyzed all data and provided inputs for interpretation of results. RG, SC, NKS and BS wrote the manuscript in consultation with other co-authors. SB conceived, planned coordinated and supervised the overall study and finalized the manuscript. All authors read and approved the final manuscript.
